# MicroLED biosensor with colloidal quantum dots and smartphone detection

**DOI:** 10.1364/BOE.478276

**Published:** 2023-02-10

**Authors:** Natalie Bruce, Francesca Farrell, Enyuan Xie, Mark G. Scullion, Anne-Marie Haughey, Erdan Gu, Martin D. Dawson, Nicolas Laurand

**Affiliations:** 1 Institute of Photonics, Department of Physics, SUPA, University of Strathclyde, Glasgow, UK; 2 Fraunhofer Centre for Applied Photonics, 99 George Street, Glasgow, UK

## Abstract

A fluorescence sensor with the capability for spatially multiplexed measurements utilizing smartphone detection is presented. Bioconjugated quantum dots are used as the fluorescent tag and are excited using a blue-emitting microLED (µLED). The 1-dimensional GaN µLED array is butt-coupled to one edge of the glass slide to take advantage of total internal reflection fluorescence (TIRF) principles. The bioassays on the top surface of the glass waveguide are excited and the resultant fluorescence is detected with the smartphone. The red, green, and blue channels of the digital image are utilized to spectrally separate the excitation light from the fluorescence for analysis. Using a biotin-functionalized glass slide as proof of principle, we have shown that streptavidin conjugated quantum dots can be detected down to a concentration of 8 nM.

## 1. Introduction

Point of care (POC) diagnostics is an expanding technological area aimed at the rapid detection of analytes without the requirement for sending biological samples to a centralized laboratory for testing. Further development of POC technology should lead to improvements in early diagnosis of diseases, better healthcare monitoring and management, which would ultimately lead to improved patient care and satisfaction [1]. Other industries where POC diagnostics could be utilized include water quality assessment [2], food safety [3], and bioterrorism agent detection [4] as this capacity would provide on-site testing.

Currently, biological assays with readings based on fluorescence, absorption or colorimetry set the standard for detection and quantification of protein biomarkers as they provide higher sensitivity and quantitative results [5]. However, these are typically processed in centralized laboratories and there is a challenge is to translate their capability to POC applications [3,5]. One way this challenge could be addressed is through the implementation of optical sensors which use the principle of total internal reflection fluorescence (TIRF) where excitation light travels through a waveguiding structure. The evanescent wave that extends beyond the waveguide excites fluorescent probes on its surface. Therefore, assays based on the immobilization of fluorescent markers can be done at the surface of the waveguide while fluorescence is monitored to infer the presence and quantity of analytes in a sample. The advantage of TIRF is that only the fluorescent probes on the surface of the waveguide are excited, resulting in lower background fluorescence and increased sensitivity [6–8].

In this paper, we introduce µLEDs as the pump source for a TIRF platform with the utilization of a smartphone for detection of the fluorescence. The waveguiding platform is a simple glass microscope slide. Crucially for miniaturization, the small emitting size of µLEDs enables efficient light coupling without any intermediate optics. This platform shares common aspects with a previously reported phototherapy and sensing device [9–11] but the waveguiding material here is glass as opposed to polydimethylsiloxane (PDMS). While more brittle than PDMS, glass has well established biofunctionalization methods that are less prone to non-specific binding, which can otherwise hamper the bio-detection. Furthermore, we explore a new detection feature that can build on the functionality of previous platforms. The CMOS sensors of smartphones respond to red (R), green (G), and blue (B) wavelengths of light, which allows for detection of changes in fluorescent intensities at different wavelengths. We take advantage of this capability to spectrally discriminate between the blue (444 nm) excitation light from the µLED array device and the red fluorescence from bio-conjugated colloidal quantum dots. [12,13]

As fluorescent markers, semiconductor colloidal quantum dots (CQDs) have several benefits such as; high quantum yield, broad absorption, high resistance to photobleaching, size-tunability resulting in their fluorescent emission covering wavelengths from the ultraviolet to infrared spectrum [14] and having lower background noise in comparison to organic dyes [15]. CQDs typically consist of a core/shell semiconductor heterostructure with ligand groups attached to the shell. The type of ligands attached to the surface of the CQDs determines which solvents the CQDs are dispersible in [16]. To be suitable for biomedical applications, CQDs are required to be hydrophilic [17,18]. CQDs are inherently hydrophobic when they are manufactured and must undergo further processing techniques such as ligand exchange or encapsulation with a polymer or silica coating to render them hydrophilic [19]. Adding proteins, enzymes or antibodies to the CQD surface allows them to be used for applications such as bioimaging or drug delivery [20] or, as in this study, sensing.

In the following section, the concept and design of the µLED-based sensor is presented. The experimental methods are detailed in section 3. The proof of principle makes use of streptavidin conjugated quantum dots that are immobilized onto biotin-functionalized glass slides. Non-specific binding is prevented through further coating with Bovine Serum Albumin (BSA). Results, including the setting of a limit of detection (LoD), obtained via analysis of the phone camera R, G and B channel responses at different CQD concentrations are presented and discussed in section 4.

## 2. Concept and design

**Fig. 1.**
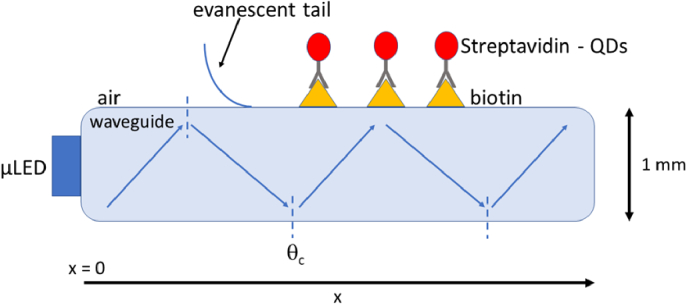
Schematic of the TIRF sensor (not to scale). For illustration it shows the path of one ray of light through the glass slide. Where the light is totally internally reflected at the glass/air interface, an evanescent wave is created, extending just above the glass surface. This evanescent wave can excite QD tags (red) bound to the biotin (yellow) on the surface of the waveguide.

The sensor platform is shown schematically in Fig. 1. A blue-emitting 444 nm GaN µLED array device that acts as the excitation source is butt coupled to the edge facet of a microscope slide (1 mm in thickness, 26 mm in width and 76 mm in length, defined as x). The µLED light is guided within the slide by total internal reflection (TIR). The evanescent waves generated by TIR are exploited to excite fluorescent tags on the surface of the slide. This fluorescence is then detected by a smartphone camera placed 90 mm above the platform to image the glass surface (see Section 3) – the fluorescence intensity being directly linked to the number of fluorescent tags immobilized on the surface. As opposed to direct fluorophore excitation, TIRF geometry minimizes autofluorescence and background noise caused by direct excitation light [6–8].

In our proof of principle demonstration, the fluorescent tags are streptavidin - conjugated colloidal quantum dots (SA-QDs) that can be immobilized on the surface of biotin-coated glass. Biotin/streptavidin is a well-understood, strong affinity binding pair that is commonly used for demonstrating the capabilities of novel biosensors [21]. Another advantage of using biotin is the possibility of using it to attach other antibodies through a process known as biotinylation to allow for further functionality and multiplexing [22]. Quantum dots (QDs) have a relatively narrow fluorescence spectrum, and their emission wavelength is judiciously chosen here to be in the red (peak at 655 nm) so the camera can more easily discriminate between fluorescence and excitation light with no crosstalk (as will be shown in section 4). This is determined from the response of the respective R, G and B channels of the smartphone sensor. The QD material used in this research is composed of a cadmium selenide (CdSe) core, surrounded by a zinc sulphide (ZnS) shell. The QDs were purchased from Invitrogen. To render the QDs hydrophilic and suitable for biological applications the manufacturer has coated the material with a polymer-shell which also provides the site for streptavidin surface conjugation.

To maximize coupling of the excitation light, the vertical diameter size of the µLED emission on the facet of the glass slide needs to be smaller than the thickness of the slide. There is less restriction in the horizontal plane of the slide, and in fact, an extended µLED emission in the shape of a line may be desirable for a homogenous “filling” of the waveguiding slide with excitation light. In contrast, if the µLED array size is too large this will impact on the power consumption, making it less viable for battery operation. Therefore, we chose a 1 × 10 array format of µLED emitters (or pixels), the dimensions of each pixel being 100 × 100 µm^2^. The spacing between each pixel is 720 µm (centre to centre) and the pixels are contacted in parallel. The µLED array is of a flip chip design with emission through the sapphire substrate. This means that the effective size of the pixels’ emission at the sapphire/glass interface, which is the relevant parameter for coupling, is increased due to light propagation through the sapphire. The substrate is 300 µm thick and therefore the size of the emission for each pixel at the substrate/glass interface is close to 300 µm [23] – i.e. below 1 mm, as required. This ensures that the geometrical coupling losses are negligible. The high numerical aperture of a slab waveguide, like the glass slide, also ensures maximisation of the angular coupling efficiency although Fresnel and scattering losses at the entrance facet limit coupling efficiency to 10% in our case (see section 3.4). We note that the µLED technology is easily scalable and negligible geometrical coupling loss can be maintained for thinner glass slides simply by adjusting the dimension of the µLED pixels.

**Fig. 2.**
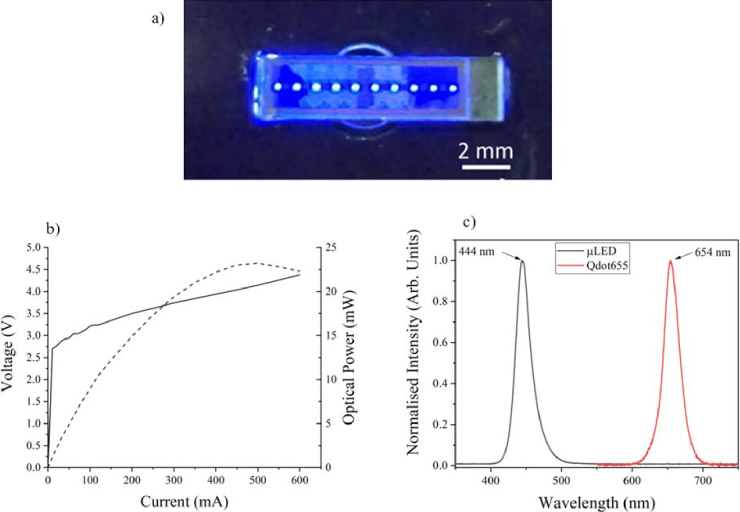
a) Images of the 1-dimensional array of µLED pixels on, with current driven at 5 mA. b) Plot of output power and voltage vs. driving current of the µLED excitation source pictured, c) Photoluminescent emission spectrum of µLED excitation source, with a peak wavelength of 444 nm and a FWHM of 25 nm and photoluminescent emission spectrum of the fluorescent tag Invitrogen Qdot655, used for the waveguide sensing, showing a peak wavelength of 654 nm and a FWHM of 27 nm

The µLED array is wire bonded to a printed circuit board (PCB). The PCB is 25 × 50 mm^2^ and has SubMiniature version A (SMA) connectors to link to an external current source for ease of demonstration. In future implementations the size of the PCB could be further reduced to incorporate a battery for portable operation. Figure 2(a) shows images of the wire bonded device with pixels on. Figure 2(b) plots the output power vs. current, and the current vs. voltage of the µLED device. The maximum output power, limited by thermal roll-over, is 23 mW reached at a drive current of 450 mA. For the sensing experiments, the µLED array was driven either at 80 mA (7.5 mW) or at 120 mA (10 mW). The peak emission wavelength of the µLED array is 444 nm with a full width half maximum (FWHM) of 25 nm, as shown in Fig. 2(c).

**Fig. 3.**
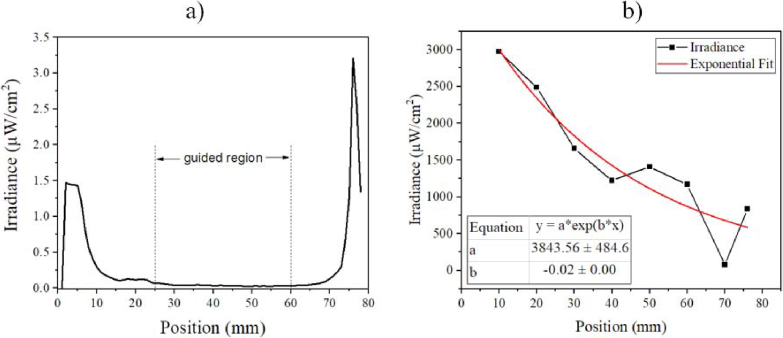
a) top-emitted irradiance values along the x-direction of the glass microscope slide b) irradiance values emitted from the end-facet of the glass waveguide to determine the propagation loss through the glass microscope slide using the cutback method.

A plot of the irradiance measured from the top of a bare glass microscope slide at different positions is shown in Fig. 3(a). The position is given by the parameter x, in mm, where x = 0 mm is the facet where the µLED array is edge-coupled (as indicated in Fig. 1). This plot gives an indication of how the light is being coupled and guided through the glass. Irradiance levels are high at the zero position (the edge-coupled facet) due to scattering from the waveguide surface and the proximity of the collecting fibre to the µLED array. The irradiance decrease between 0 mm and 20 mm is mainly caused by the unguided light scattered out of the slide – a smaller contribution is from the diffraction in the plane of the waveguide, as the coupled light spreads and fills the slide’s cross-section. Although it can be seen from 25 mm to 60 mm along the x – axis that the measured top irradiance is low and appears mostly constant; there is a small decreasing trend caused by propagation losses through the glass material. An increase in the irradiance at the end of the glass microscope slide can also be seen and this is due to increased light exiting the waveguide whilst being scattered by surface roughness at the edges of the glass. The measurement of the top irradiance in the transverse direction (y), not shown, has a similar trend with edge effect and a low, almost constant value between 7 mm to 15 mm. It is in this region of the glass slide (25 mm < x < 60 mm and 7 mm < y < 15 mm), where the light from the µLED array is being guided, and the SA-QDs are best placed on the top surface for excitation in this area, without excessive background light being present. The propagation losses of the light guided in the x direction were measured using the cut-back method (see section 3). Figure 3(b) plots the results; the loss through the glass is calculated to be 0.2 cm^-1^. These results show that the level of guided excitation light in this region, can be considered relatively homogeneous within +/-30%. This measurement was then repeated after biofunctionalization of the glass slide. While the top irradiance is then higher (because of the biomolecules coating the glass surface) the irradiance is still homogeneous within 30%. The propagation loss is also higher, estimated at 0.36 cm^-1^.

## 3. Materials and methods

### 3.1 Biofunctionalisation process and surface immobilization

**Fig. 4.**
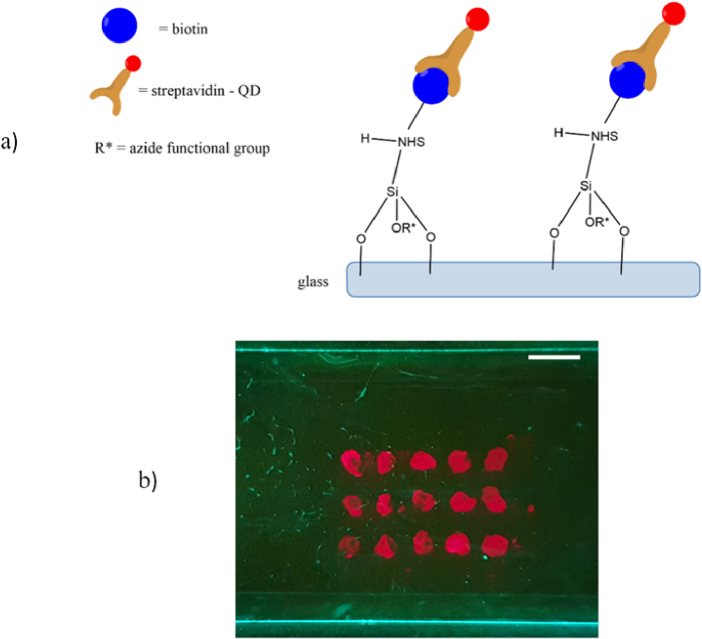
a) Schematic of the bonding structure of the biotin to streptavidin quantum dots on the glass microscope slide following silianization from APTES and functionalization with NHS-biotin. b) Image of a glass microscope slide showing the configuration of the quantum dot regions pipetted onto the surface, scale bar is 5 mm.

The surface of the glass slides used in this work were functionalized using the following method. Plasma treatment of the silica glass microscope slides (dimensions 76 × 26 × 1 mm^3^) was performed to activate functional surface bonds and enable further functionalization with N-Hydroxysulfosuccinimidobiotin as explained below; a schematic of this bonding/functionalization is shown in Fig. 4(a). The slides were placed in a plasma reactor (Diener Atto Plasma Surface Treatment Machine) for approx. 30 s @ 20 W, 1 mbar pressure. The glass was then treated with 3-aminopropyltriethoxysilane (2% v/v in de-ionized water) for 2 hours. The glass slides were then rinsed in de-ionized water (DI), dried with a nitrogen aspirator gun, and placed in an oven at 80 °C for 5 minutes. The N-Hydroxysulfosuccinimidobiotin (NHS-Biotin) powder (Sigma Aldrich Biotin-NHS, Water-Soluble) was mixed with phosphate buffer saline (PBS), (Fischer BioReagents, Phosphate Buffered Saline, 1X Solution, pH 7.4), to a concentration of 1 mg/ml. 1 ml of the solution was pipetted onto the surface of the glass and left to react with the surface. Following this, excess NHS-Biotin solution was rinsed using PBS and DI water respectively and then dried with the aspirator. 4% BSA, (Thermo Fisher, Blocker BSA (10X) in PBS), was used to reduce the non-specific binding on the glass surface, which was placed in solution for 2 hours. Once removed from the BSA solution, the glass slides were rinsed again with PBS and DI water and dried.

Once dried, the glass slides were ready to test the immobilization of SA-QDs (Invitrogen, Qdot 655 streptavidin conjugate). SA-QDs were diluted from their original 1 µM solution using 4% BSA in varying concentrations from 100 nM to 1 nM. One glass slide per concentration was prepared, 0.5 µL was micro pipetted onto the surface 15 times in a 3 × 5 array format, as shown in Fig. 4(b). The SA-QDs were left in contact with the surface for 30 seconds after deposition and then rinsed. The glass slides were rinsed by immersion in 4% Tween 20 (Fisher BioReagents), followed by PBS then DI water; this was repeated 3 times. The glass slides were left to dry fully before images were captured. To confirm that the SA-QDs present after rinsing were due to attachment to biotin rather than non-specific binding to the glass surface, this process was also conducted for glass slides prepared using the same method but omitting treatment with biotin. It was confirmed through the absence of SA-QDs on the glass surface following rinsing that non-specific binding was negligible in our experiments.

### 3.2 Smartphone measurements

**Fig. 5.**
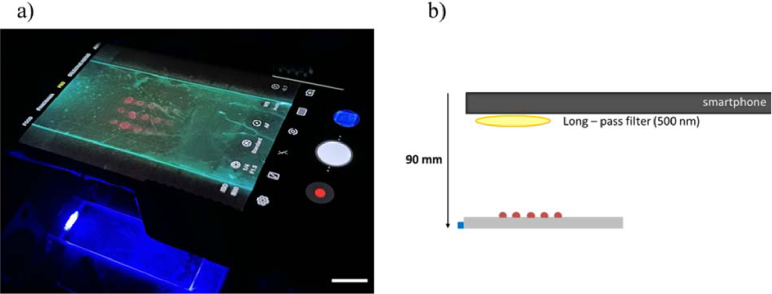
a) Photograph of the smartphone being used to take an image of fluorescence produced by the QDs on the glass surface while being illuminated with the µLED, scale bar is 20 mm and b) a schematic of the smartphone detection system.

To measure the intensity output of the SA-QD regions, the glass microscope slide was edge-coupled to the µLED array (see section 2). The µLED array was operated with a driving current of 120 mA for limit of detection measurements and to compare the effect of different exposure times on the measured intensity. To determine the effect of a lower driving current on intensity output, the glass slides were also imaged with the driving current set to 80 mA. Each microscope slide contained 15 regions of one SA-QD concentration. To obtain an intensity value for each concentration, the fluorescence intensity of these 15 regions was averaged. A Samsung Galaxy S9 smartphone was used as a detector for the intensity output of the SA-QDs as shown in Fig. 5(a). Figure 5(b) shows a schematic detailing the experimental set-up, where the smartphone is placed at a fixed height of 90 mm above the sensor membrane, with a long pass filter with a cut off wavelength of 500 nm, placed over the camera of the smartphone.

The camera settings for the smartphone are outlined in Table 1 and were set using the in-built pro mode in the camera software. Images were saved as JPEG files.

**Table 1.** Samsung Galaxy S9 Smartphone Pro Mode Camera Settings

Sensor Setting	Value

ISO	800
Exposure Time	50 ms, 250 ms, 1000 ms
Zoom	2x
Aperture	f/1.5

The JPEG images were used for data analysis, and intensity values were extracted using Image J software. RGB images of the waveguides were converted to a greyscale RGB stack separating the pixel information of the red, green, and blue channels. The regions where quantum dots had been deposited were selected and the software analysed the pixel data over the selected areas to give a mean pixel intensity for that area. The mean pixel intensities for the 15 quantum dot areas were then averaged to give the fluorescence intensity value at a given SA-QD concentration. Each of these measurements were furthermore done in triplicate (uncoupling the glass slide from the µLED and recoupling the slide between each measurement) and averaged to give a measure of repeatability of the intensities for a specific concentration and determine the standard deviations for each concentration. This process was carried out for the R, G, B channels as well as the combined grey scale (RGB) images.

### 3.3 Top irradiance measurements

To determine the best place to drop cast the SA-QDs with minimal background interference, the irradiance emitted from the top of a blank glass microscope slide was measured. The µLED array was driven at 120 mA current while a microscope slide was butt-coupled to the sapphire surface. A fibre optic was held at 4 mm above the glass slide. An Avantes spectrometer (Avantes, Starline AvaSpec – 2048L) was calibrated and used to measure the spectral irradiance of the guided mode emitted from the top surface of the glass slide. Measurements were taken at 1 mm intervals along the full length (x direction) of the 76 mm glass slide. Once all the measurements had been collected, the data was integrated over the desired wavelengths (400–500 nm) and the irradiance of the evanescent wave emitted from the glass was plotted against the position. This was repeated for the y – direction at the 45 mm position of the glass slide.

### 3.4 Propagation losses

An estimate of the transmitted power loss through the waveguide was obtained following the cutback method [24]. The glass waveguide was butt-coupled to the µLED array while being driven at 120 mA. The spectral irradiance emitted from the end fact of the waveguide was measured using an optical fibre connected to a spectrometer (Avantes, Starline AvaSpec – 2048L). The initial measurement was taken at the original length of the glass waveguide at 76 mm. The length of the glass was then cutback by 6 mm, and the spectral irradiance was measured at 70 mm. This cutback process repeated at 10 mm intervals until the glass waveguide was 20 mm long. Spectral irradiance data was then integrated over the desired wavelengths (400 - 500 nm) and irradiance values were plotted against position. To determine an estimation of the guiding losses, the plot was fitted using an exponential decay rate. The plot was also extrapolated to x = 0 to give a value for the irradiance coupled into the glass slide of 3.8 mW/cm^2^; given the cross section of the slide this corresponds to an optical power close to 1 mW (*P_ (guiding irradiance)_).* Given this estimated power coupled into the waveguide and knowing the LED optical power (*P_(_*_*µ* *LED)*_) is 10 mW, a coupling efficiency of 10% as stated in section 2, is obtained as per Eq. 1.


(1) $$\begin{array}{c} n=\;\frac{P\;{\;}_{\;\left(guiding\;irradiance\right)}}{P_{\;\left(\mu LED\right)}\;} \end{array}$$

### 3.5 Characterizing quantum dots

SA-QDs (Invitrogen, Qdot 655 streptavidin conjugate) were diluted using PBS to a give a solution concentration of 16 nM to assess the photoluminescent optical properties of the quantum dots. The solution was in a 1 cm x 1 cm microcuvette, placed in a 4 – port cuvette holder (Thorlabs, CVH100) and excited using a 532 nm laser (Thorlabs, 532 nm DPSS, DJ532-10) with an optical output power of 10 mW. A spectrometer was used to collect the photoluminescent spectrum of the SA-QDs set at 90 degrees to the laser diode to avoid interference from the excitation source. Using the same solution, the absorbance spectrum was measured using a spectrophotometer (Thermo Scientific, GENESYS 30 Visible Spectrometer). Photoluminescent spectrum of the Invitrogen Qdot 655 streptavidin conjugated quantum dots is shown in Fig. 2(c) with a peak emission of 654 nm and FWHM of 27 nm. The absorbance spectrum has a peak of 616 nm.

### 3.6 Characterizing µLED array properties

For the measurements shown in Fig. 2, the µLED array emission was collected and focused onto a detector using a pair of aspheric condenser lenses (AC4532-A, Thorlabs) with a diameter of 45 mm, a focal length of 32 mm and numerical aperture of 0.612. The collection efficiency of the Lambertian µLED array emission by this set-up is 21%. Optical output power was measured using a calibrated silicon photodetector (Thorlabs, S121C); the LED current was increased incrementally while the supplied voltage and optical power were recorded. Results are shown in Fig. 2(b). The µLED array spectra were measured using the same optical set up described previously, with the photoluminescent spectrum recorded using a fibre-coupled Ocean Optics Spectrometer (OceanOptics, USB 4000). The photoluminescent spectrum is shown in Fig. 2(c). The peak emission wavelength is 444 nm with a FWHM of 25 nm.

## 4. Results and discussion

### 4.1 Mean pixel intensity

**Fig. 6.**
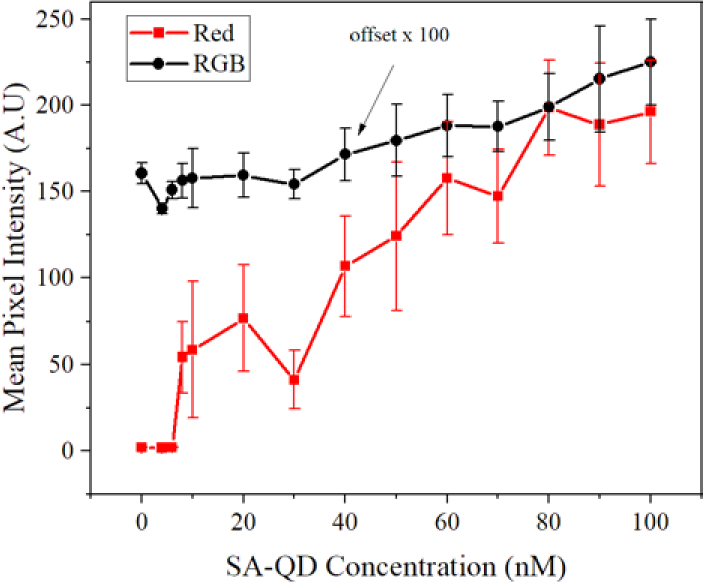
Results from a glass microscope slide with streptavidin conjugated QDs, showing that the mean pixel intensity increases with concentration. A comparison between the greyscale RGB pixel data (offset by a factor of 100) and the red pixel data indicating that changes in intensity for the red channel are caused by the increasing concentration of QDs.

Figure 6 shows the mean pixel intensity of the R channel, for an exposure time of 250 ms at a LED drive current of 120 mA, plotted against the concentration of the SA-QDs. It can be seen that as the concentration of the SA-QDs increases, the mean pixel intensity of the R channel increases. The sensor response is approximately linear between 8 nM and 80 nM and saturates at higher concentrations, indicating at this exposure time, the detector of the smartphone has saturated. The lowest SA-QD concentration that yields a signal above the noise floor detection limit is 8 nM. The LoD is defined here as the concentration giving an intensity reading 3 times the standard deviations above the intensity of a sample with 0 nM of SA-QDs present. Below this concentration (8 nM), the sample regions were not bright enough to be detected by the smartphone sensor and were below the noise floor. A possible explanation is that the expected response of the sensor is sigmoidal, hence the drop at low concentrations may be steeper and the experimental conditions do not permit a high enough resolution in the concentrations below such levels. Also, there may not be enough biotin present on the surface of the glass to have the SA-QDs efficiently binds to the surface with an adequate intensity level to be detected. The data point (30 nM) for the red signal intensity is lower than expected. This is an outlier attributed to fabrication challenges of the glass waveguide in repeatedly creating even biological layers at each step of the process. We note that the LoD is camera specific, and a calibration would need to be done if using another mobile phone/camera. Figure 6 also displays the greyscale (RGB) data, which has been offset by multiplying its intensity by a factor of 100. Unlike the R channel, the RGB response is non-zero at a zero concentration. This signal is from the scattered light from the µLED array; whilst the blue light has been filtered out the luminescence of the µLED array has a non-negligible tail at green wavelengths, which is being picked up by both the B and G response of the camera. However, the value at 0 and overall trend of the R channel indicate that the red intensities captured by the smartphone are occurring solely from the emission of the quantum dots and are not influenced by the green or blue channel contributions – the sensor therefore fully discriminates the fluorescence from the µLED array light. Detection of concentrations as low as 80 pM have been reported in previous research using alkyl ligand quantum dots [10], however the latter used alkyl ligand CQDs that were simply drop casted with no bio-detection unlike the water soluble SA-QDs that were used in this research and are necessary for biological applications [16]. As the previous research did not encompass bio-detection, all the quantum dots drop cast onto the membrane will have stayed in situ.

### 4.2 Effect of the driving current

**Fig. 7.**
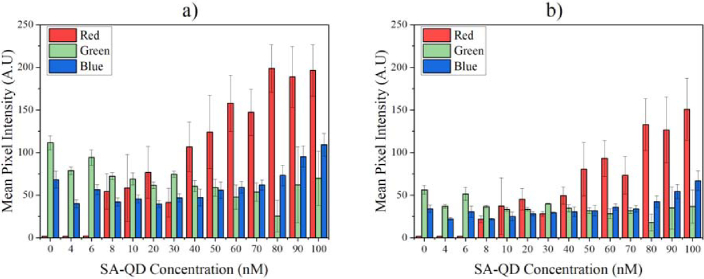
a) and b): mean pixel intensities of the red, green, and blue channels at an exposure time of 250 ms, driven at a current of 80 mA and 120 mA respectively. The red channel intensity increases with concentration of the SA-QDs, while the green and blue channels indicate the level of scattering from the µLED light as it travels through the glass waveguide.

Figure 7(a) and (b) show the mean pixel intensities of the R, G, and B channels from the smartphone images at an exposure time of 250 ms, with the driving current being 80 mA and 120 mA respectively. Both graphs indicate that above 8 nM the SA-QD intensity for the red channel increases with concentration (as was seen in Fig. 6). Increasing the current helps to achieve a higher mean pixel intensity value for each channel and reduces the standard deviations for repeatability. Although the intensity values detected by the smartphone sensor have increased for each channel, the LoD remains in practice the same at 8 nM whether the current is driven at 80 mA or 120 mA. Increasing (reducing) further the drive current (hence optical power) may lead to a noticeable reduction (increase) of the LoD. However, thermal effects and long-term stability may raise challenges that were not investigated in this work.

As stated previously, the R channel detects the SA-QDs independently with no influence from the pump light being detected, even with an increase in the amount of overall light the smartphone sensor detects. The behaviours are similar at the two drive currents for both B and G channels. The trend is for the intensity of the G channel to decrease slightly with increasing QD concentration. This is consistent with the fact that the G channel only encompasses light from the LED, which is being depleted at higher QD concentrations (due to the absorption by the QDs). The B channel is similar but the response increases at the highest QD concentrations – possibly because the B channel has a non-negligible response to red photon and/or because of an increase in scattered LED light (this is possibly seen in the G response as well). However, the magnitude of these trends is significantly lower than the changes in the R response.

### 4.3 Effect of exposure times

**Fig. 8.**
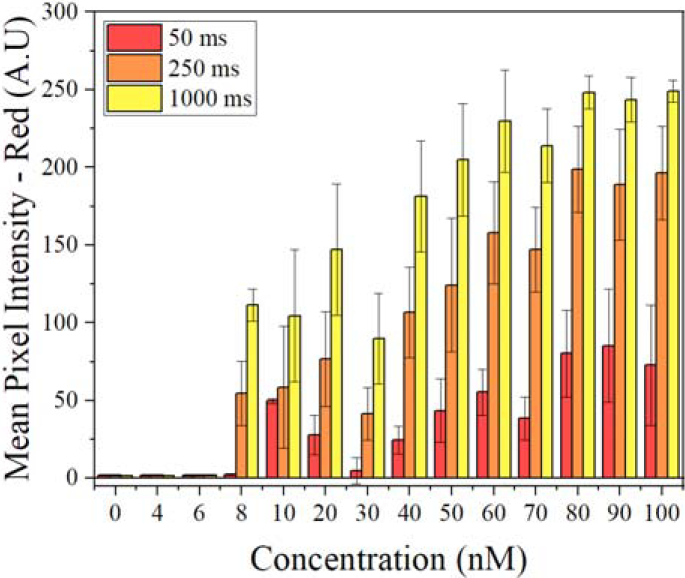
Mean pixel intensities at different exposure times plotted against QD concentration while the µLED is driven at 120 mA.

Three different exposure times were compared to determine the effect of increased time capturing the image on the mean pixel intensities of the red, green, and blue sensor channels; these results are shown in Fig. 8. The expected effect of increasing exposure time would be to increase the signal acquired by the red pixels and improve the LoD for lower concentrations of SA-QDs. At 50 ms the LoD is 10 nM, upon increasing the exposure time to 1000 ms the LoD is improved to 8 nM. This increase in mean intensity from 50 ms to 1000 ms for SA-QD regions at 8 nM is 46 times higher. With this improvement in detection comes a change in the noise floor for the detector. The noise floor at 50 ms is 2 counts in comparison to 80.9 counts at 1000 ms exposure. Below 8 nM, the smartphone sensor is unable to detect a significant intensity count of red photons, highlighting that for this application the limitations of detection for this smartphone and experimental set-up have been reached. While increasing the exposure time can increase detection of lower SA-QD concentrations, conversely, decreasing the exposure time to 50 ms will allow for a larger range of concentrations to be detected. At a 100 nM concentration, 50 ms exposure time, the mean pixel intensity was found to be 72 counts; this is less than half the maximum intensity value that can be detected with the smartphone sensor.

## 5. Conclusion

This paper demonstrates that a µLED array-based evanescent waveguide platform can be used as a relatively simple fluorescent biosensor with smartphone detection. µLEDs enable efficient light coupling without any additional optics and scaling down the thickness of the waveguide platform, while maintaining efficiency, is possible. These factors contribute positively towards development of a compact POC device that has the potential to be battery operated. Importantly, the RGB response of the detector sensor can discriminate between fluorescence and excitation light by appropriate choice of the µLED and QD emission wavelengths, meaning that simple JPEG images can be taken for readings. While this report details the use of one type of smartphone, it is probable that different phone models could require different optimisation for limits of detection of sensing experiments. This could be made possible through development of a multi-platform app designed for imaging and sensing allowing for use among many different users. Streptavidin-functionalized QDs were utilized as the fluorophores in this demonstration where the core shell material contains toxic and non-environmentally friendly chemicals such as cadmium. Further work making use of materials such as carbon quantum dots or indium phosphide materials that avoid these materials would make for better alternatives in the future. However, these materials are still in their infancy in terms of photoluminescent properties. In turn, a detection limit of 8 nM at an exposure of 250 ms has been established. By demonstrating that functionalized amine ligand QDs can be detected using a smartphone it provides a promising basis for the platform to be used as a compact biosensor for POC.

## Data Availability

Data underlying the results presented in this paper are available in Ref. [25].
